# Aneurisma da artéria isquiática persistente: relato de caso de tratamento endovascular

**DOI:** 10.1590/1677-5449.008617

**Published:** 2018

**Authors:** Dominique Rodas Costa, Ronald José Ribeiro Fidelis, Vanessa Prado dos Santos, Cícero Fidelis, Carlos Alberto Silveira Alves, José Siqueira de Araújo

**Affiliations:** 1 Universidade Federal da Bahia – UFBA, Serviço de Cirurgia Vascular do Complexo Hospitalar Universitário Professor Edgard Santos, Salvador, BA, Brasil.; 2 Universidade Federal da Bahia – UFBA, Instituto de Humanidades, Artes e Ciências Professor Milton Santos, Salvador, BA, Brasil.; 3 Universidade Federal da Bahia – UFBA, Faculdade de Medicina da Bahia, Departamento de Cirurgia, Salvador, BA, Brasil.

**Keywords:** aneurisma, procedimentos endovasculares, persistência da artéria isquiática, extremidade inferior

## Abstract

A persistência da artéria isquiática é uma anomalia vascular congênita rara cuja principal complicação é a dilatação aneurismática. O quadro clínico pode incluir sintomas decorrentes da dilatação arterial e da isquemia, causada por trombose ou embolização distal. O tratamento dessa afecção rara conta com opções diversas que abrangem desde a ligadura do aneurisma até a correção endovascular. O presente relato descreve o caso de uma paciente do sexo feminino com queixa de tumoração pulsátil na região glútea. Foi encaminhada ao serviço de referência e realizou angiotomografia, que evidenciou persistência completa da artéria isquiática bilateralmente, com dilatação aneurismática à esquerda. A paciente foi submetida a tratamento endovascular do aneurisma, através de punção contralateral, com implante de dois *stents* revestidos com manutenção da perviedade distal da artéria. A manutenção da perviedade é particularmente importante nos casos da forma completa dessa variação anatômica. A paciente cursou com boa evolução.

## INTRODUÇÃO

A persistência da artéria isquiática é uma anomalia vascular rara, com incidência estimada em 0,01 a 0,06%^1^^,^ 2. Consiste em uma anomalia congênita na qual a artéria isquiática persistente (AIP) se situa em continuidade com a artéria ilíaca interna após a emergência das artérias glútea superior e pudenda interna^3^. A persistência da artéria isquiática pode ser classificada como forma completa ou incompleta. Na forma completa, ela continua até a artéria poplítea, sendo a artéria dominante da extremidade^1^. Na forma incompleta, a artéria isquiática persistente é hipoplásica, sendo o sistema femoral dominante, e pode haver comunicação entre a AIP e a artéria poplítea através de ramos colaterais^1^.

A dilatação aneurismática é uma das alterações que podem ocorrer na AIP, afetando cerca de 44 a 48% dos casos^1^^,^ 2. A etiologia da dilatação aneurismática não está clara; pode estar relacionada a repetidos traumas, predisposição a aterosclerose e hipoplasia do tecido conectivo^1^. Sintomas clínicos variáveis, com casos de isquemia e dilatação aneurismática, são relatados na literatura^1^^-^ 6.

Este trabalho relata um caso de persistência bilateral da artéria isquiática com dilatação aneurismática à esquerda. Trata-se de um caso diagnosticado e tratado através da técnica endovascular pelo Serviço de Cirurgia Vascular no Complexo Hospitalar Universitário Professor Edgard Santos/Hospital Ana Nery na cidade de Salvador (BA).

## DESCRIÇÃO DO CASO

Paciente do sexo feminino, 76 anos, com história de sensação de pulsação anormal, indolor, na região glútea esquerda por aproximadamente cinco anos. Nos cinco meses anteriores, percebeu pulsação na genitália e dor na região glútea esquerda associada aos movimentos, como ao se sentar. Negava história de trauma local, claudicação intermitente ou outras queixas nos membros inferiores. Não relatava outras comorbidades, apenas passado de tabagismo havia mais de 20 anos. Procurou atendimento em sua cidade de origem, onde foi inicialmente avaliada com ultrassom de partes moles, que identificou dilatação vascular medindo 2,7 cm no tecido celular subcutâneo da região glútea esquerda. Após a realização do exame ultrassonográfico, a paciente foi encaminhada para avaliação no Serviço de Cirurgia Vascular do Complexo Hospitalar Universitário Professor Edgard Santos/Hospital Ana Nery.

A paciente foi admitida no ambulatório do serviço e, ao exame físico, apresentava massa pulsátil com cerca de 5 x 7 cm de diâmetro no quadrante inferolateral da região glútea esquerda, indolor, com frêmito à palpação. Ao exame vascular, todos os pulsos estavam presentes e simétricos.

**Figura 1:**
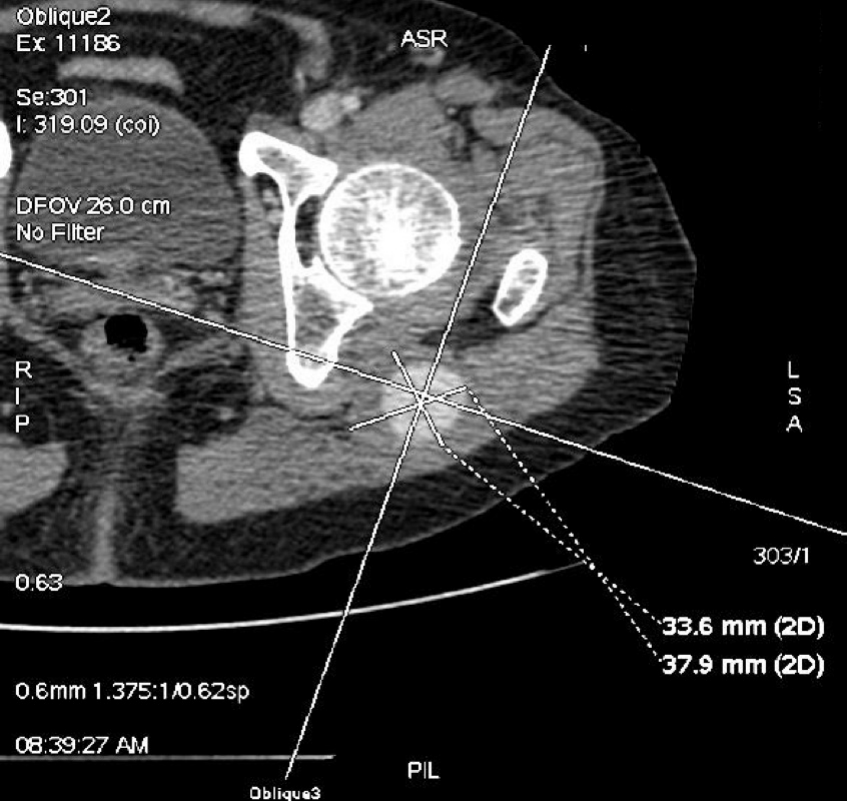
Angiotomografia em corte axial evidenciando o diâmetro (37,9 x 33,6 mm) do aneurisma da artéria isquiática persistente.

Diante do quadro clínico e dos exames físico e ultrassonográfico, formulou-se a hipótese diagnóstica de aneurisma da AIP. Foi solicitada uma angiotomografia de aorta abdominal e de membros inferiores, que demonstrou aorta abdominal de calibre normal e trajeto levemente tortuoso em segmento infrarrenal, com ateromatose parietal difusa e sem estenoses. Evidenciou-se também perviedade das artérias ilíacas comuns, internas e externas, destacando-se hipoplasia das ilíacas externas e predominância das ilíacas internas, que continuavam bilateralmente com a artéria isquiática na face posterior de ambas as coxas. Observava-se, ainda, ectasia da AIP à esquerda, com aneurisma fusiforme e trombo mural, com maior diâmetro de 3,7 x 3,3 cm e extensão de 6,4 cm (Figuras 1 e 2).

**Figura 2:**
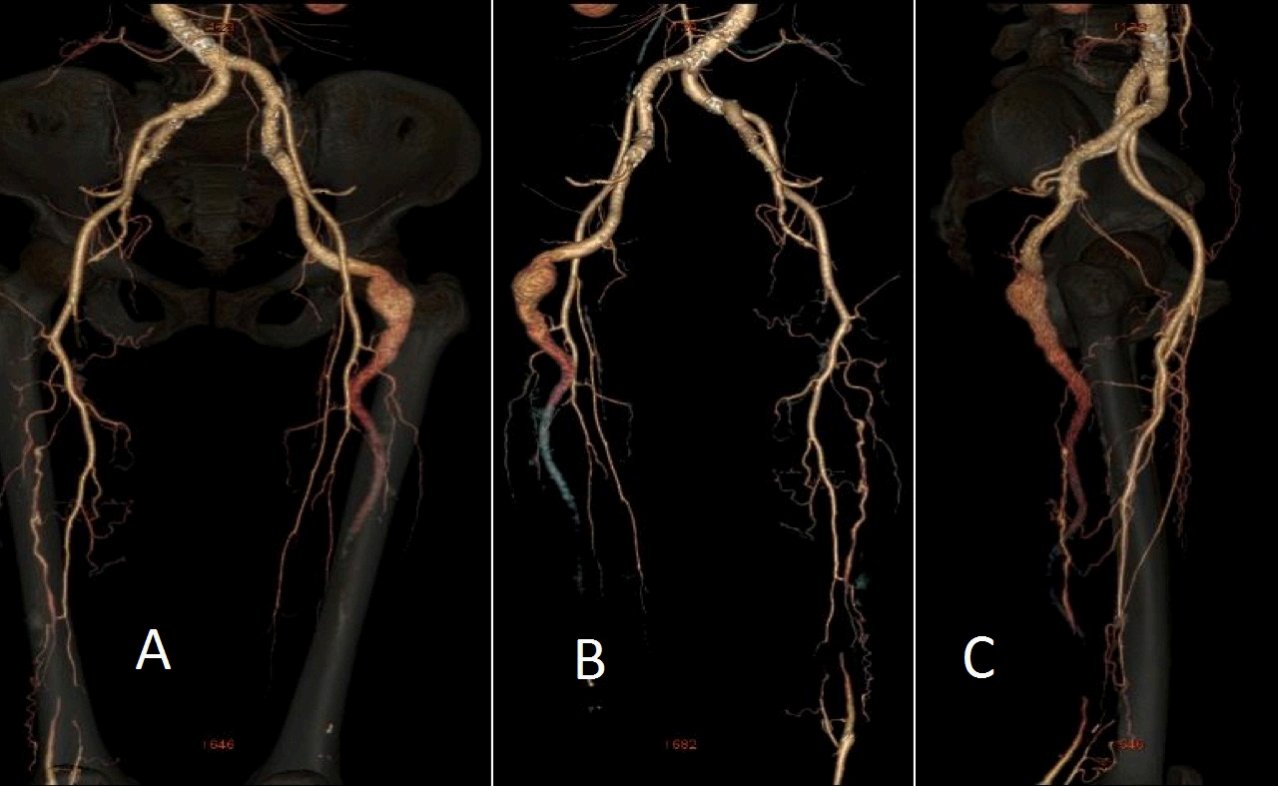
Angiotomografia evidenciando persistência bilateral da artéria isquiática com formação aneurismática à esquerda: imagens de reconstrução na visão anterior (A), posterior (B) e lateral (C).

**Figura 3:**
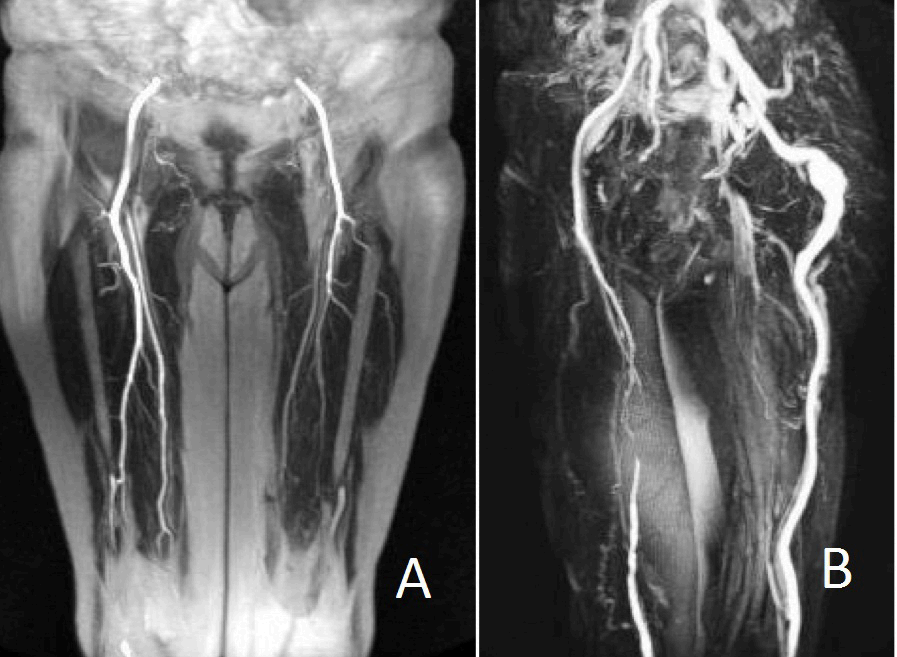
Angiorressonância: hipoplasia de artérias femorais superficiais e profundas (A) e artérias isquiáticas dando origem às artérias poplíteas, o que caracteriza a forma completa de persistência da artéria isquiática bilateralmente. Dilatação aneurismática fusiforme da artéria isquiática persistente à esquerda (B).

Foi realizada uma angiorressonância para detalhar as artérias dos membros inferiores. Havia hipoplasia de ambas as artérias femorais superficiais e as artérias femorais comuns e profundas estavam com calibres preservados. A artéria isquiática continuava, bilateralmente, com a artéria poplítea, caracterizando a forma completa dessa anomalia vascular. As artérias infrageniculares encontravam-se pérvias e com calibres normais (Figura 3).

**Figura 4:**
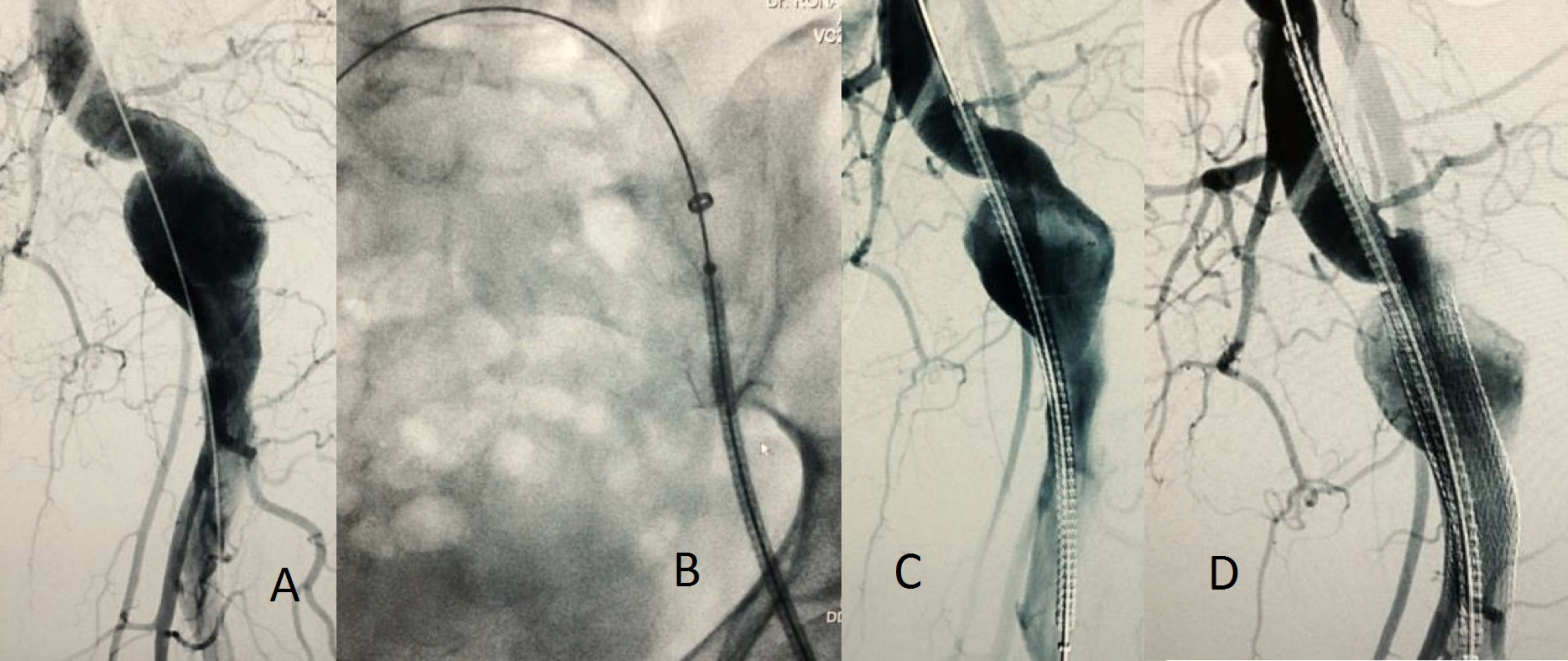
Tratamento endovascular do aneurisma da artéria isquiática persistente à esquerda: (A) angiografia através de acesso contralateral pela artéria femoral comum direita; (B) e (C) posicionamento do primeiro *stent* revestido (distal) 13 x 10 mm. (D) posicionamento do segundo *stent* revestido (proximal) 11 x 10 mm.

A paciente foi então encaminhada para tratamento endovascular do aneurisma sintomático da AIP esquerda, visto que mantinha quadro de dor local progressiva e constante. O tratamento proposto foi a correção endovascular do aneurisma de AIP com dois *stents* revestidos (Viabahn® - Gore®), para exclusão completa do saco aneurismático e manutenção da patência da artéria isquiática. Por se tratar de um caso de persistência completa, a AIP fornece o principal aporte sanguíneo para o membro inferior esquerdo, tendo continuidade com a artéria poplítea. O tratamento endovascular do aneurisma de AIP à esquerda foi iniciado através de acesso contralateral pela artéria femoral comum direita, sendo realizado o implante do *stent* revestido distal (13 x 10 mm), seguido do implante do segundo *stent* revestido proximal (11 x 10 mm), com bom aspecto (Figuras 4 e 5).

**Figura 5:**
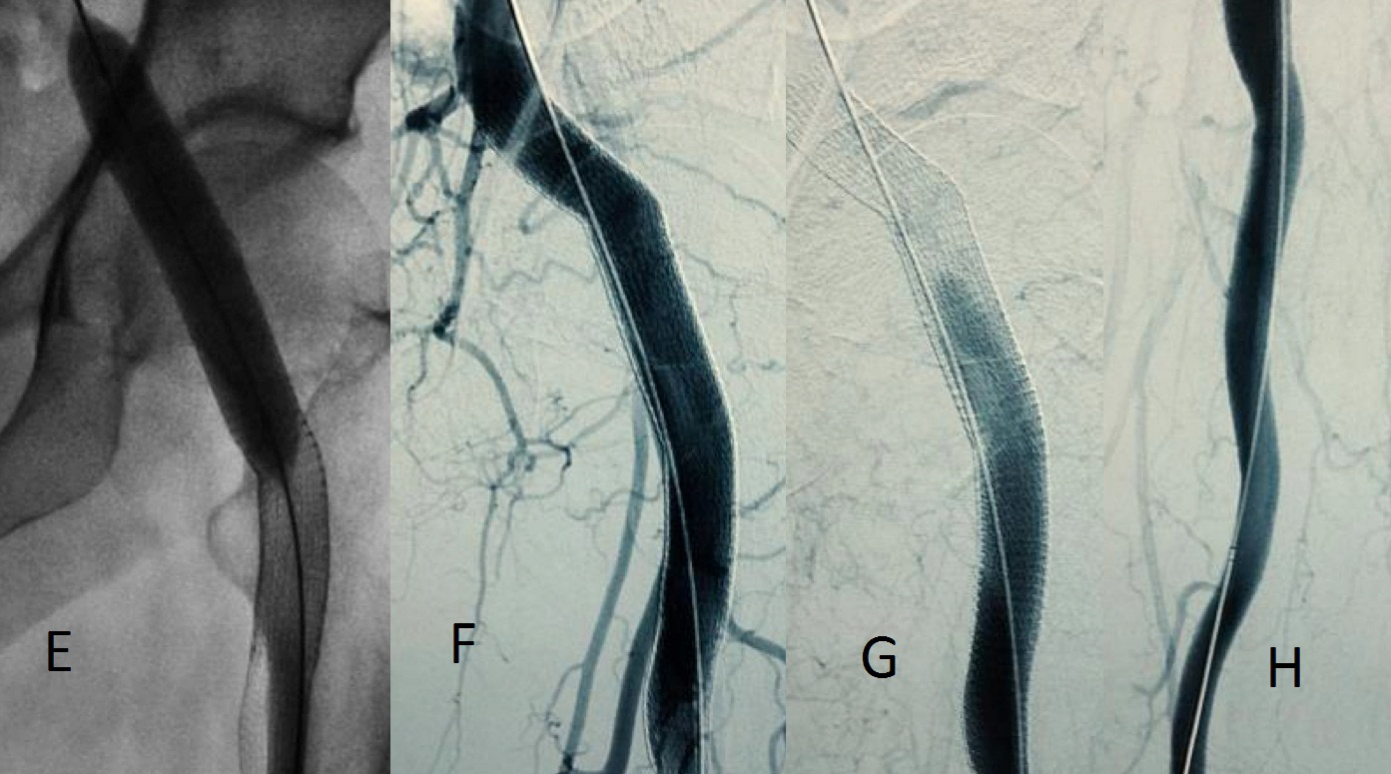
Tratamento endovascular do aneurisma da artéria isquiática persistente: (E) balonamento proximal, distal e em conexão dos *stents* revestidos com balão 12 x 60 mm; (F) e (G) angiografia de controle evidenciando exclusão do aneurisma e ausência de vazamento; (H) angiografia de artéria isquiática distalmente ao tratamento do aneurisma mostrando perviedade.

**Figura 6:**
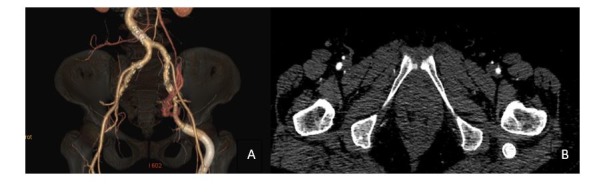
(A) Angiotomografia realizada após o tratamento endovascular, com reconstrução tridimensional em anteroposterior, mostrando *stents* revestidos na artéria isquiática persistente pérvios e sem vazamentos; (B) corte axial da angiotomografia evidenciando regressão completa da dilatação aneurismática na artéria isquiática persistente esquerda.

Não houve intercorrência durante a correção endovascular do aneurisma de AIP, e a paciente se manteve estável durante todo o procedimento, recebendo alta hospitalar dois dias após o procedimento, com melhora da dor e ausência de massa pulsátil na região glútea esquerda. A paciente concordou em participar do relato de caso através do termo de consentimento livre e esclarecido. Após um ano do tratamento endovascular, a paciente mantinha-se assintomática em acompanhamento ambulatorial no serviço, com pulsos distais presentes. Nesse período, foi realizada uma angiotomografia de controle, que evidenciou *stents* revestidos pérvios e sem vazamentos, com regressão completa da dilatação aneurismática na AIP esquerda (Figura 6).

## DISCUSSÃO

A artéria isquiática é uma continuação da artéria ilíaca interna, sendo a principal responsável pelo suprimento vascular para o membro inferior durante o período embrionário^3^. No terceiro mês de desenvolvimento embrionário, a artéria isquiática regride e o sistema femoral torna-se o principal responsável pelo fluxo sanguíneo do membro inferior^3^^,^ 7. Apesar de a AIP ser uma ocorrência rara na população, a possibilidade de isquemia e dilatação aneurismática torna o diagnóstico diferencial importante na prática clínica^7^.

O quadro clínico pode variar desde o paciente assintomático, com queixa de claudicação intermitente ou presença de massa pulsátil, até quadros de emergência como isquemia aguda de membro inferior ou aneurisma roto^2^^,^ 8 ^,^ 9. Uma revisão da literatura realizada por Van Hooft et al. mostrou que 80% dos doentes eram sintomáticos à época do diagnóstico^2^. Ao exame físico, o sinal de Cowie, que consiste na ausência do pulso femoral com pulso poplíteo palpável, foi descrito em poucos casos^2^^,^ 10.

Em alguns trabalhos, a arteriografia vem sendo descrita como principal método diagnóstico nos casos de AIP^1^^-^ 3. No entanto, um estudo realizado com uso de angiotomografia mostrou que esse método também pode ser utilizado na avaliação da AIP^11^. Jung et al. avaliaram 307 angiotomografias e encontraram AIP em seis pacientes (1,63% dos exames), com dilatação aneurismática em dois casos^1^.

O tratamento da AIP varia de acordo com a apresentação clínica do doente. Os pacientes assintomáticos, sem dilatação, provavelmente podem ser acompanhados regularmente através dos exames físico e de imagem^1^. Nos quadros de isquemia de membro inferior, os casos relatados mostraram a realização de pontes femoropoplíteas ou femorodistais com a utilização da veia safena magna como substituto arterial^4^^,^ 6 ^,^ 9 ^,^ 12 ^-^ 15. A utilização de prótese de dácron e a angioplastia de AIP também são descritas na literatura^16^^,^ 17. Nos casos de claudicação, o acompanhamento sem tratamento cirúrgico foi indicado em alguns casos^3^^,^ 13 ^,^ 18. Nas persistências incompletas, a ligadura da AIP, sem revascularização, também já foi descrita^3^^,^ 19. Uma revisão da literatura com 146 casos de AIP encontrou cinco casos de amputações maiores^2^. Nas dilatações aneurismáticas, os tratamentos descritos mostram as possibilidades de ligadura do aneurisma através da cirurgia convencional ou endovascular pela embolização, com ou sem necessidade de revascularização, devendo ser avaliado se a persistência é completa ou incompleta^2^^,^ 6 ^,^ 13 ^,^ 19.

Na nossa paciente, optamos pelo tratamento endovascular com uso de dois *stents* revestidos, por se tratar de persistência completa, com bom resultado. Há relato na literatura de um paciente de 53 anos com aneurisma da AIP de 7 cm associado à isquemia distal, em que os autores descrevem o tratamento endovascular através da utilização de dois *stents* revestidos (Hemobahn®)^5^. Apesar da raridade do diagnóstico, o tratamento endovascular do aneurisma da AIP pode evitar potenciais complicações relacionadas ao acesso cirúrgico, devido à proximidade do nervo ciático e veia^7^. Quanto à possibilidade de fratura do *stent* revestido, um estudo realizado com aneurismas da artéria poplítea tratados através dessa técnica revelou um índice de fratura de 16,7%, sem influência significativa na patência do *stent* 20.

O aneurisma da AIP é uma doença rara, que requer um alto índice de suspeição para seu diagnóstico. O tratamento passa por uma ampla gama de possibilidades, na dependência do quadro clínico das características anatômicas de cada caso. O estudo completo da circulação pélvica e dos membros inferiores é recomendável para o planejamento do tratamento. Os avanços da cirurgia endovascular podem trazer contribuições para o tratamento da AIP, com sucesso terapêutico, como no caso aqui relatado.
