# Supplementary material for: Aneurisma da artéria isquiática persistente: relato de caso de tratamento endovascular
Source: J Vasc Bras. 2018 Jan-Mar;17(1):49–54. [Article in Portuguese] doi: 10.1590/1677-5449.008617 (PMC5990260; doi:10.1590/1677-5449.008617)
Supplement: Translation

# Translation: Persistent sciatic artery aneurysm: case report of endovascular treatment

**Authors:** Dominique Rodas Costa, Ronald José Ribeiro Fidelis, Vanessa Prado dos Santos, Cícero Fidelis, Carlos Alberto Silveira Alves, José Siqueira de Araújo

**Affiliations:** 1 Universidade Federal da Bahia – UFBA, Serviço de Cirurgia Vascular do Complexo Hospitalar Universitário Professor Edgard Santos, Salvador, BA, Brasil.; 2 Universidade Federal da Bahia – UFBA, Instituto de Humanidades, Artes e Ciências Professor Milton Santos – IHAC, Salvador, BA, Brasil.; 3 Universidade Federal da Bahia – UFBA, Faculdade de Medicina da Bahia, Departamento de Cirurgia, Salvador, BA, Brasil.

**Keywords:** aneurysm, endovascular procedures, persistent sciatic artery, lower extremity

## Abstract

A persistent sciatic artery is a rare congenital vascular anomaly. The most common complication is aneurysm. Clinical presentation may include symptoms resulting from arterial dilatation and ischemia caused by thrombosis or embolization. There are diverse options for treatment of this rare condition, ranging from ligature of the aneurism to endovascular repair. This report describes the case of a female patient complaining of a pulsating mass in the left buttock. She was referred to a Vascular Service where an angiotomography showed complete bilateral persistence of the sciatic artery, with an aneurysm of the left sciatic artery. The aneurysm was treated with endovascular techniques, via a contralateral approach, with placement of two stent grafts and preservation of distal patency. Patency is particularly important in cases of this anatomical variation in which the persistent sciatic artery is complete. This patient recovered well.

## INTRODUCTION

Persistence of the sciatic artery is a rare vascular anomaly, with an estimated incidence of 0.01 to 0.06%.^1^^,^ 2 It is a congenital anomaly in which a persistent sciatic artery (PSA) exists as a continuation of the internal iliac artery after emergence of the superior gluteal and internal pudendal arteries.^3^ Persistence of the sciatic artery can be classified as complete or incomplete. In the complete form, the artery continues to the popliteal artery and is the dominant artery in the limb.^1^ In the incomplete form, the persistent sciatic artery is hypoplasic, the femoral system is dominant, and there may be communication between the PSA and the popliteal artery via collateral branches.^1^

One related pathology that can be associated with PSA is aneurysmal dilatation, present in around 44 to 48% of cases.^1^^,^ 2 The etiology of aneurysmal dilatation is unclear; but it may be related to repeated traumas, predisposition to atherosclerosis, and hypoplasia of connective tissues.^1^ Clinical symptoms vary, with cases of ischemia and aneurysmal dilatation reported in the literature.^1^^-^ 6

This paper describes a case of bilateral sciatic artery persistence with an aneurysmal dilatation of the left sciatic artery. The case was diagnosed and treated using endovascular techniques at the Vascular Surgery Service of the Complexo Hospitalar Universitário Professor Edgard Santos/Hospital Ana Nery, in the city of Salvador, BA, Brazil.

## CASE DESCRIPTION

The patient was a 76-year-old female with a history of an abnormal, painless, pulsing feeling in the left gluteal region, with onset approximately 5 years previously. During the 5 months preceding diagnosis she had noticed pulsation in the genitalia and pain in the left gluteal region, associated with movements such as sitting down. She reported no history of local trauma, intermittent claudication, or other complaints affecting the lower limbs. She did not report other comorbidities, just a smoking habit that she had quit more than 20 years previously. She had sought care in her home town, where she had been examined with ultrasound of soft tissues, identifying a vascular dilation measuring 2.7 cm in the subcutaneous tissues of the left gluteal area. After this ultrasonographic examination, the patient had been referred for assessment to the Vascular Surgery Service at the Complexo Hospitalar Universitário Professor Edgard Santos/Hospital Ana Nery.

The patient was admitted by the vascular clinic and, on physical examination, exhibited a painless pulsating mass with a diameter of approximately 5 x 7 cm in the inferolateral quadrant of the left gluteus, with thrill on palpation. Vascular examination found all pulses present and symmetrical.

In view of this clinical status and the findings of physical and ultrasonographic examinations, a diagnostic hypothesis of PSA aneurysm was formulated. Angiotomography of the abdominal aorta and lower limbs was ordered, showing a normal caliber abdominal aorta with a slightly tortuous course in the infrarenal segment, with diffuse parietal atheromatosis, but free from stenosis. The examination also showed that the common, internal, and external iliac arteries were all patent, with notable hypoplasia of the external iliacs and predominance of the internal iliacs, which continued bilaterally together with the sciatic artery along the posterior surfaces of both thighs. The left PSA was dilated, with fusiform aneurysm and mural thrombus, with a largest diameter of 3.7 x 3.3 cm and an extension of 6.4 cm (Figures 1 and 2).

**Figure 1:** Axial angiotomography showing the diameter (37.9 x 33.6 mm) of the persistent sciatic artery aneurysm.

**Figure 2:** Angiotomography showing bilateral persistent sciatic arteries with aneurysmal formation involving the left sciatic artery: reconstructed images from anterior (A), posterior (B) and lateral views (C).

Magnetic resonance angiography was used to illustrate the details of the lower limb arteries. There was hypoplasia of both superficial femoral arteries, while the common and deep femoral arteries were of normal caliber. The sciatic arteries continued, bilaterally, to the popliteal arteries, characterizing the complete form of this vascular anomaly. The infragenicular arteries were patent with normal calibers (Figure 3).

**Figure 3:** Magnetic resonance angiography: hypoplasia of superficial and deep femoral arteries (A) sciatic arteries giving origin to popliteal arteries, characterizing the complete form of sciatic artery persistence bilaterally. Fusiform aneurysmal dilatation involving the left persistent sciatic artery (B).

The patient was referred for endovascular treatment of the symptomatic aneurysm of the left PSA, since she was still suffering localized, progressive, and constant pain. The treatment proposed was endovascular repair of the PSA aneurysm using two covered stents (Viabahn® - Gore®) to completely exclude the aneurysm sac and preserve patency of the sciatic artery. Because the patient had a complete form of PSA, this artery was responsible for the principal blood supply to the left lower limb, running to the popliteal artery. Endovascular treatment of the PSA aneurysm was initiated via a contralateral access through the right common femoral artery with implantation of the distal covered stent (13 x 10 mm), followed by implantation of the proximal covered stent (11 x 10 mm), with good final appearance (Figures 4 and 5).

**Figure 4:** Endovascular treatment of the aneurysm of the left persistent sciatic artery: (A) angiography via a contralateral access through the right common femoral artery; (B) and (C) placement of first (distal) covered stent, 13 x 10 mm, (D) placement of second (proximal) covered stent, 11 x 10 mm.

**Figure 5:** Endovascular treatment of the persistent sciatic artery aneurysm: (E) ballooning of proximal and distal covered stents and at connection using a 12 x 60 mm balloon; (F) and (G) control angiography showing exclusion of the aneurysm and absence of leaks; (H) angiography of the sciatic artery distal of the aneurysm repair, showing patency.

There were no intraoperative complications during the process of endovascular repair of the PSA aneurysm and the patient remained stable throughout the entire procedure. She was discharged from hospital 2 days after the procedure, with improvement of the pain and no pulsating mass in the left gluteal region. The patient agreed to this case report, signing a free and informed consent form. One year after the endovascular treatment, she was still asymptomatic and in outpatients follow-up at the service, with distal pulses present. During this period, a control angiotomography was conducted, showing that the covered stents were patent and free from leaks, with complete regression of the aneurysmal dilatation of the left PSA (Figure 6).

**Figure 6:** (A) Angiotomography performed after endovascular treatment, with anteroposterior three-dimensional reconstruction, showing covered stents in the persistent sciatic artery, patent and free from leaks; (B) axial angiotomography showing complete regression of the aneurysmal dilatation of the left persistent sciatic artery.

## DISCUSSION

The sciatic artery is a continuation of the internal iliac artery and is the primary vascular supply to the lower limb in the embryo.^3^ During the third month of embryonic development, the sciatic artery regresses and the femoral system becomes the primary supplier of blood flow to the lower limb.^3^^,^ 7 Although PSA is a rare occurrence in the population, the possibility of ischemia and aneurysmal dilatation means that its differential diagnosis is important in clinical practice.^7^

Clinical status can range from asymptomatic patients, through complaints of intermittent claudication or presence of pulsating masses, to emergency cases, such as acute lower limb ischemia or ruptured aneurysms.^2^^,^ 8 ^,^ 9 A literature review conducted by Van Hooft et al. showed that 80% of patients were symptomatic at the time of diagnosis.^2^ The Cowie sign, consisting of absent femoral pulse with palpable popliteal pulse, was described as present at physical examination in a small number of cases.^2^^,^ 10

Some authors describe arteriography as the principal diagnostic method for PSA cases,^1^^-^ 3 but a study conducted using angiotomography showed that this method can also be used to investigate PSA.^11^ Jung et al. examined 307 angiotomographies and found PSA in six patients (1.63% of examinations), in two of whom there was aneurysmal dilatation.^1^

The treatment for PSA varies according to clinical presentation. Asymptomatic patients, without dilatation, can probably be managed with regular monitoring using physical and imaging examinations.^1^ Cases with lower limb ischemia that have been described were treated with femoropopliteal or femorodistal bypasses, using the great saphenous vein as a substitute artery.^4^^,^ 6 ^,^ 9 ^,^ 12 ^-^ 15 Grafts with dacron prostheses and PSA angioplasty have also been described in the literature.^16^^,^ 17 Monitoring, with no surgical treatment, has been recommended in some cases with claudication.^3^^,^ 13 ^,^ 18 In incomplete persistent arteries, ligature of the PSA without revascularization has also been described.^3^^,^ 19 A review of 146 cases of PSA in the literature identified five cases of major amputation.^2^ Treatments described for cases with aneurysmal dilatation include ligature of the aneurysm by conventional surgery or endovascular embolization, with or without the need for revascularization, depending on the result of investigation to determine whether persistence is complete or incomplete.^2^^,^ 6 ^,^ 13 ^,^ 19

With our patient, we decided to use endovascular techniques to conduct treatment with two covered stents, because the persistence was complete, with good results. There is a report in the literature of a 53-year-old patient with a 7 cm PSA aneurysm associated with distal ischemia, in which the authors describe endovascular treatment with two covered stents (Hemobahn®).^5^ Despite the rarity of the diagnosis, endovascular treatment of PSA aneurysms can avoid potential complications related to surgical access, due to proximity to the sciatic nerve and vein.^7^ With regard to the possibility of fracture of a covered stent, a study of popliteal artery aneurysms treated using this technique found a fracture rate of 16.7%, without significant effects on stent patency.^20^

Aneurysm of a PSA is a rare disease and a high degree of clinical suspicion is needed for diagnosis. There are a wide range of treatment possibilities, depending on the clinical status and anatomic characteristics of each case. A thorough study of pelvic and lower limb circulation is recommended for treatment planning. The advances achieved in endovascular surgery have contributions to make to PSA treatment, offering the possibility of therapeutic success, as in the case described here.
